# New Insight into the Ground State of FePc: A Diffusion Monte Carlo Study

**DOI:** 10.1038/s41598-017-01668-6

**Published:** 2017-05-17

**Authors:** Tom Ichibha, Zhufeng Hou, Kenta Hongo, Ryo Maezono

**Affiliations:** 1 School of Information Science, JAIST, Nomi, Ishikawa Japan; 2 National Institute of Materials Science, Tsukuba, Ibaraki Japan; 3 PRESTO, JST, Kawaguchi, Saitama Japan

## Abstract

We have applied DMC to evaluate relative stability of the possible electronic configurations of an isolated FePc under *D*_4*h*_ symmetry, considering some fixed nodes generated from different methods. They predict *A*_2*g*_ ground state consistently, supporting preceding DFT studies, with confidence overcoming the ambiguity about exchange-correlation (XC) functionals. By comparing DMC with several XC, we clarified the importance of the short-range exchange to describe the relative stability. We examined why the predicted *A*_2*g*_ is excluded from possible ground states in the recent ligand field based model. Simplified assumptions made in the superposition model are identified to give unreasonably less energy gain for *A*_2*g*_ when compared with the reality. The state is found to have possible reasons for the stabilization, reducing the occupations from an unstable anti-bonding orbital, avoiding double occupation of a spatially localized orbital, and gaining exchange energy by putting a triplet spin pair in degenerate orbitals.

## Introduction

Iron(II) Phthalocyanine (FePc) attracts recent interests for its potentials in spintronics^1–3^ because it possesses the strong magnetic anisotropy as a molecular magnet^4^. It has been reported the anisotropy can be controlled by surrounding environments of molecules, such as ligands, polymorphs of molecular crystal structures etc.^2, 5, 6^. These environments tune the electronic configuration of the central transition metal element, affecting its magnetic anisotropy. Identifying the electronic configuration under given environments is therefore the most essential starting point for further understandings and applications of the magnetic anisotropy of these compounds, stimulating intensive studies in this direction. An earlier study^7^ reported its spin multiplicity being in between *S* = 1~2^7^. Later, Dale *et al*.^8^ performed magnetic susceptibility measurements of *β*-FePc, reporting that the system takes *S* = 1 in the range, *T* = 1.25~20 K. Since then, the possible configurations within *S* = 1 have been of the interests. Even under this constraint, no consensus has been established about its ground state configuration.

Experimentally, the most common targets are molecular crystals with lamination angles, *ϕ* = 44.8° (*β* phase/most stable structure) and *ϕ* = 26.5° (*α* phase/quasi stable). The most stable *β* phase has been the main interest until *α*-FePc was reported^9, 10^ to exhibit ferromagnetic transition at *T*_*c*_ = 5.6 K, while *β* remains paramagnetic until above 1 K. The *α* phase then attracts broader interests for its ferromagnetism with the spin anisotropy lying within its molecular plane with unquenched orbital angular momentums^2, 11^. Under practical samples in experiments, the ground state configuration of the *α* phase has been reported as *E*_*g*_(*a*), forming a consensus^1, 4, 11^.

Apart from intensive discussions on possible factors affecting the configuration in practical samples, such as inter-complex interactions in crystals^6, 12^, the spin-orbit coupling etc.^2, 13^, it is a reasonable option to start considering the simplest situation, namely, an isolated, highly symmetric molecule. Electronic structure calculations using DFT (density functional theory) have hence been made for the isolated molecule. However, even without the spin-orbit coupling, theoretical predictions have never dropped in a consensus as described below. We hence target the most simplified question, “ *what is the ground state electronic configuration for the ideal isolated FePc within the non*-*relativistic framework*?”. Most of DFT studies so far predict *A*_2*g*_ ground state^6, 14, 15^, while a recent study^15^ reports that the prediction actually depends on the choice of exchange-correlation (XC) functionals and Gaussian basis set level, getting both *A*_2*g*_ and *B*_2*g*_ as the possibility. The same conclusion is actually confirmed in the present study, getting mainly *A*_2*g*_, but sometimes *B*_2*g*_ and even *E*_*g*_(*a*) depending on XC.

Another complementary approach is the ligand field framework^2, 16, 17^. It is capable to be applied to the *D*_4*h*_ isolated molecule, predicting *E*_*g*_(*b*) ground state for the ligand parameter choice for *α* phase. (When the spin-orbit coupling is taken into account, it predicts a hybrid state between *E*_*g*_(*b*) and *B*_2*g*_ as the ground state, latter of which is predicted as the first excited state when without the coupling. The magnetic anisotropy is predicted being perpendicular to [within] the molecular plane for *E*_*g*_(*b*) [*B*_2*g*_]. In the hybrid state, it is within the plane despite the dominant state is *E*_*g*_(*b*)^2﻿^). The original ligand field model for *D*_4*h*_ requires three ligand parameters to identify the possible ground state, where *A*_2*g*_ still remains as a possibility^16^, not conflicting with *ab initio* DFT predictions. In a recent work^17^, however, the possible ground state is specified by reduced two parameters and *A*_2*g*_ has disappeared from the possibility, leading to an apparent contradiction to DFT predictions. The reduction of the freedom of parameters occurs when they employ the superposition model^18^ under some assumptions.

The present study targets to investigate the apparent discrepancy about *A*_2*g*_ ground state between *ab initio* and ligand field model^17^ approaches. Blocked by the ambiguity of predictions due to XC, this discrepancy has not well been addressed and investigated so far. To prevent the ambiguity, we applied (fixed-node) diffusion Monte Carlo (DMC)^19^ to calibrate the XC dependence^20–22^. Although CASSCF (complete active space self-consistent field) seems a natural choice of trial nodes appropriate for describing the multi-reference nature in transition metals, a recent work suggests it is not necessarily the best for iron complexes, finding some DFT trial nodes are better^23^. Hence we tried several trial nodes generated from DFT with M06, M06L, and M06-2X functionals as well as CASSCF (Computational details in the present study are given in Supplementary Information).

We have found all the DMC predictions support *A*_2*g*_ ground state, being consistent with most of previous DFT calculations. The apparent contradiction with the ligand field model^17^ can be explained by further considering the validation of the assumptions in the superposition model. The assumptions turn out not capable to capture the stabilizing mechanisms of *A*_2*g*_, which are clarified by the orbital shape/occupation analysis by the present study.

## System

We investigate the ground state electronic configuration of an isolated FePc molecule under *D*_4*h*_ symmetry. While there are two preceding studies reporting the possible geometry, the one from X-ray diffraction of *β* phase^24^ and the other from DFT geometry optimization applied to an isolated complex^25^, we used the latter for the present calculation (See Supplementary Information). The system accommodates six electrons in 3*d*-shells from Fe ion. Within the constraint of spin triplet, *S* = 1, there are four possible configurations labeled as,

1 $$\begin{array}{lllll}\,{A}_{2g} & : & {({a}_{g})}^{\uparrow \downarrow }{({e}_{g})}^{\uparrow \uparrow }{({b}_{2g})}^{\uparrow \downarrow } & = & {({d}_{{z}^{2}})}^{\uparrow \downarrow }{({d}_{xz,yz})}^{\uparrow \uparrow }{({d}_{xy})}^{\uparrow \downarrow },\\ \,{B}_{2g} & : & {({a}_{g})}^{\uparrow }{({e}_{g})}^{\uparrow \downarrow \uparrow \downarrow }{({b}_{2g})}^{\uparrow } & = & {({d}_{{z}^{2}})}^{\uparrow }{({d}_{xz,yz})}^{\uparrow \downarrow \uparrow \downarrow }{({d}_{xy})}^{\uparrow },\\ {E}_{g}(a) & : & {({a}_{g})}^{\uparrow }{({e}_{g})}^{\uparrow \downarrow \uparrow }{({b}_{2g})}^{\uparrow \downarrow } & = & {({d}_{{z}^{2}})}^{\uparrow }{({d}_{xz,yz})}^{\uparrow \downarrow \uparrow }{({d}_{xy})}^{\uparrow \downarrow },\\ {E}_{g}(b) & : & {({a}_{g})}^{\uparrow \downarrow }{({e}_{g})}^{\uparrow \downarrow \uparrow }{({b}_{2g})}^{\uparrow } & = & {({d}_{{z}^{2}})}^{\uparrow \downarrow }{({d}_{xz,yz})}^{\uparrow \downarrow \uparrow }{({d}_{xy})}^{\uparrow }.\end{array}$$

Any occupations to ${d}_{{x}^{2}-{y}^{2}}$ are excluded from the possibility because the orbital makes a strong σ*-coupling with neighboring ligands to get unstabilized.

## Results

### DMC results

**Figure 1:**
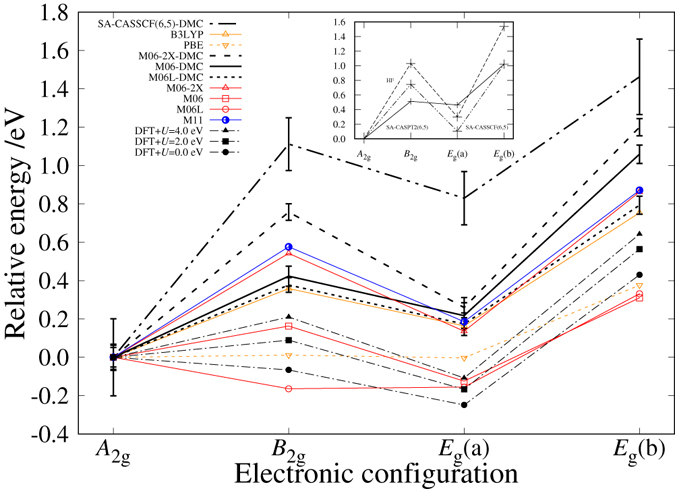
The predictions of some *ab initio* methods. This graph shows the predictions of CASSCF-DMC, DFT-DMC, HF, CASSCF, CASPT2, and some DFT calculations about the relative stability among the four electronic configurations.

Predictions of the relative stability among the states are shown in Fig. 1, compared with each other among DMC, CASSCF, and DFT. A pronounced feature of the present DMC predictions (shown as bold lines) is the ‘N-shaped’ dependence [the lowest *A*_2*g*_ and a dip at *E*_*g*_(a)]. Since CASSCF-DMC is reported to be ‘*not best*’ in the nodal quality sense^23^, we also performed several DFT-DMC results using M06, M06L, and M06-2X functionals for further confirmation. We see that all the DMC give the N-shape with the lowest energy by *A*_2*g*_, being consistent with each other. For M06L, it is interesting to see that ‘non-N-shape’ at DFT level turns to ‘N-shape’ at fixed-node DMC level. Among the DFT-DMC, the M06 is found to give *variationaly best* nodal surfaces, making total energies of each state lower than those by M06L and M06-2X by around ~0.4 eV in average. We cannot make unfortunately the variational comparison between DFT-DMC and CASSCF-DMC because the latter is an all-electron simulation, while the former a pseudo potential one (See Supplementary Information for computational details).

### Inconsistency of DFT predictions

While most of preceding DFT reported *A*_2*g*_ predicted as the ground state, we clearly see here again that the prediction indeed depends on the choice of XC, as already pointed out by several authors^13, 15, 26^. Assuming the results by M06-DMC being the more reliable prediction, we can focus on its ‘N-shaped’ dependence being a target property to be reproduced by properly selected XC. We see that the B3LYP-DFT prediction is quite similar to the M06-DMC one, which would support the validation of DFT predictions by this functional to some extent. We could identify that the short-range exchange contribution is quite essential to reproduce the ‘N-shape’, as discussed below.

Looking at DFT+*U* results^27^, we see that the ‘N-shape’ gets recovered as *U* increases, corresponding to taking into account the exchange component more. The same tendency can also be confirmed by the results^28^ using a hybrid functionals, M06. In the series of M06 in Fig. 1, the Fock exchange contribution increases as M06L(0%) → M06(27%) → M06-2X (54%), and it approaches to the ‘N-shaped’ dependence. M11 in Fig. 1 also includes the Fock exchange contribution but it is separated into short- and long-range components. The comparison between M11 and M06-2X clearly shows us which component matters: The two functionals both contain the Fock exchange at almost the same fraction in total, 54% (M06-2X) and 42.8% (M11), but they are different in the their fractions of long-range component, 54% (M06-2X) and 100% (M11). Almost the same ‘N-shape’ for both implies that the improvement in M11 from M06-2X (taking into account the long-range exchange components) does not so matter in the present case to reproduce the ‘N-shape’. Though the improvement is known to affect a lot in reproducing long-ranged natures such as van der Waals interactions^29^, what matters in the present case seems rather the short-range component, or to say the self-interaction nature of the exchange.

## Discussions

### Short-range exchange in DFT

The implication of the importance of the short-range exchange component is in accordance with the fact that the increase of *U* in DFT+*U* enhances the ‘N-shape’. This would be encouraging to omit the costly evaluation of long-range exchange when one investigates more realistic periodic molecular crystals. Since the exchange interaction represents the energy gain by the orbital overlap only for spin-parallel pairs, it would critically affect when we estimate the energy differences between the states with different spin-pair configurations. While in DMC such factors are taken into account by default, DFT treatments require the special attention to choose XC functional so that it could include enough short-range exchange component to describe proper trends in energy differences.

As mentioned in the introduction, it is difficult to find such experiments those are exactly realizing the isolated/non-relativistic molecular system to be compared with the present theoretical estimations. Photoelectron spectroscopy of FePc in gas phase^30^ can be the nearest case for this purpose. Another earlier spectroscopy was reported^31^ for the molecule in solvent, concluding *A*_2*g*_ as its ground state configuration because only this configuration can explain the observed shape of spectrum. A recent spectroscopic study^30^ indicates B3LYP agrees reasonably well with experiment while PBE does not. This is rather consistent with our trend in Fig. 1, where PBE fails to reproduce the N-shape while B3LYP with exchange components can do it. These consistencies would support our conclusion that the N-shape by DMC describes the correct order of the energetic stability for each of the electronic configurations.

### Exchange v.s. Correlation effects

Static correlation describing multi-reference nature is captured by CASSCF beyond HF, while dynamical correlation describing hybridization between iron and ligands by CASPT2, though the second-order perturbation theory is well known to overestimate the dynamic one for open shell system^32^. The corresponding change in Fig. 1 can then be identified as a consequence of the correlation effect, which decreases relative energy differences among the configurations making the N-shape less pronounced. In contrast, exchange makes the shape more pronounced. We could then identify the ratio, *γ* = (correlation/exchange), would be a factor to dominate the N-shape. Ratios for M06L and PBE get larger owing to incomplete inclusion of exchange part, and then the shape would is less pronounced. A comparison between M06 and B3LYP would lead to an assumption that the shape would be dominated by the ratio rather than the absolute intensity of exchange, because their inclusion percentages of Fock exchange term are almost the same (27% and 25%) but their shapes considerably differ from each other. This might be attributed to the difference of the *ratio γ*.

Table 1 lists CASSCF expansion coefficients, which can be roughly considered extent of multi-reference nature. According to these values, *A*_2*g*_ has a less multi-reference nature than the others. This is just corresponding to the fact that the *amplitude* of the dependence in Fig. 1 gets reduced by CASSCF compared with HF. The reduction can be explained by the larger energy stabilizations for each configuration than in *A*_2*g*_, those are due to the static correlations by more enhanced multi-reference nature, leading to smaller energy differences between each configuration than in HF.

**Table 1:** The multi determinants calculated by SA-CASSCF for each electronic configurations.

State	Coefficient	3*d*_*xy*_	3*d*_*xz*_	3*d*_*yz*_	${{\boldsymbol{d}}}_{{{\boldsymbol{z}}}^{{\bf{2}}}}$	${{\boldsymbol{d}}}_{{{\boldsymbol{x}}}^{{\bf{2}}}-{{\boldsymbol{y}}}^{{\bf{2}}}}$
*A*_2*g*_	0.985	↑↓	↑	↑	↑↓	
*B*_2*g*_	0.924	↑↓	↑↓	↑	↑	
	−0.345	↑	↑	↑↓	↑↓	
*E*_*g*_(a)	0.941	↑↓	↑↓	↑	↑	
	−0.239	↑↓	↑	↑	↑	↓
*E*_*g*_(a)	0.778	↑	↑↓	↑	↑↓	
	−0.382	↑	↑↓	↑	↑	↓

This table shows the occupation of *d* shell of each determinant and its coefficient. ↑ and ↓ means up spin and down spin respectively. Only the dominating determinants are listed up, whose coefficient’s absolute values are higher than 0.2.

Though we cannot make clear statements on how the ratio *γ* in XC affects the qualities of nodal surfaces in fixed-node DMC, but we can see that the *amplitude* of the dependence in Fig. 1 gets larger in the order as M06L → M06 (variationally best among DFT-DMC) → M06-2X getting closer to CASSCF-DMC. This trend can be regarded to be driven by the decrease of *γ* for the nodal surface generation by DFT. The *location* of CASSCF-DMC at the end of this trend would be consistent with the preceding report^23^ in the sense that the incomplete inclusion of (short-range) correlations in CASSCF leads to the diminished *γ* for nodal surface generation.

### Comparison with superposition model

**Figure 2:**
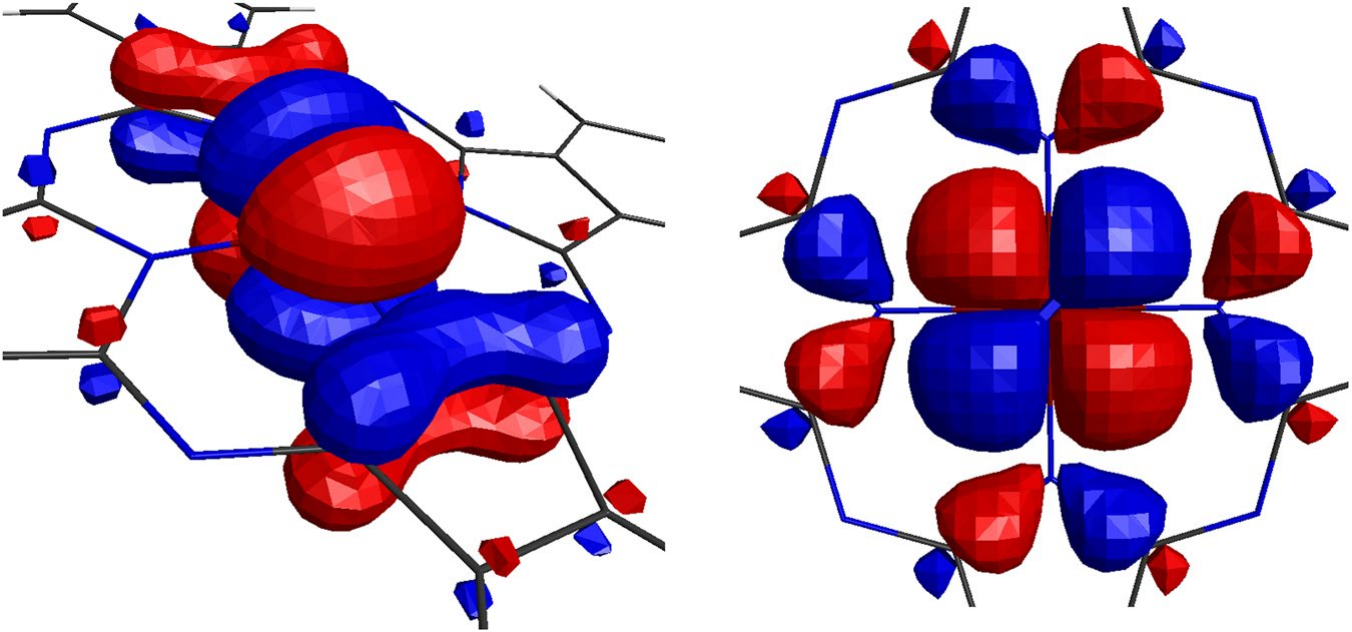
The figures of 3d orbitals. *e*_*g*_ (left) and *b*_2*g*_ (right) orbitals are evaluated by SA-CASSCF(6,5). ﻿(Drawn by MacMolPlt^﻿﻿35^).﻿

The present DMC again supports the preceding DFT predictions of ^3^ *A*_2*g*_ ground state. Such a possibility is, however, not permitted by the recent ‘ligand field’-based model^17^, as shown in Fig. 3 in their paper. We can explain this apparent contradiction by examining the assumptions made in the models. Key is the reduction of number of ligand parameters to specify the ground state in the model. Original ligand field model for *D*_4*h*_ requires three parameters, (*D*_*q*_, *D*_*s*_, *D*_*t*_), to specify it, as in the studies^2, 16^, where ^3^ *A*_2*g*_ is still in the possibility for the ground state (*e*.*g*. in Fig. 2 in the paper by Miedema *et al*.^16^). In the recent study by Kuz’min *et al*.^17^, however, the ground state is specified only by two ligand parameters under the constraint, *D*_*t*_ = (2/35) · 10*D*_*q*_, denying the ^3^ *A*_2*g*_ possibility. The constraint comes from the more restricted geometry than *D*_4*h*_ under the assumptions by the superposition model^18^: It assumes that (a) only the nearest neighboring ligands (N in this case) are considered, (b) ligand fields are specified only via bond-length dependence, (c) Total ligand field is a superposition of each ligand contribution, (d) Each contribution is assumed being axially symmetric around the intervening bond between the ligand and central element (Fe-N in this case). The assumption makes the system perfect square being able to put a Fe-N bond as *x* (or *y*) axis to get more constraint, as explained in Kuz’min *et al*.^17^. We thus notice that the ^3^ *A*_2*g*_ possibility has disappeared by this assumption.

The assumption, especially (d), seems to be easily refuted by the *ab initio* analysis for realistic treatments of actual ligands. The left panel in Fig. 2 shows the shape of *e*_*g*_ evaluated by SA-CASSCF(6,5), being obviously not the case of the assumption (d). We can give further possible explanations why the model assumption makes the ^3^ *A*_2*g*_ unstabilized more than the *ab initio* prediction: From the occupations given in Eq. (1), we notice that ^3^ *A*_2*g*_ is stabilized by reducing the number of the *e*_*g*_ occupation. As seen in the left panel (Fig. 2), the orbital forms strong *π*-coupling with neighboring N, spreading along the molecular plane. The orbital has the opposite phase to that of Fe, forming an unstabilized anti-bonding state. The ^3^ *A*_2*g*_ stabilizes itself by reducing such an energy loss made by the *e*_*g*_ occupation. This stabilization mechanism cannot be captured by the assumptions of superposition model at all. Another mechanism would be captured by the right panel of Fig. 2, where *b*_2*g*_ orbital stabilizes itself by leaking its distribution toward outer ligands, which is not taken into account in the model. The model then describes the spurious confinement for the electrons in *b*_2*g*_ orbital, getting its energy level increased than the *ab initio* estimation. This would also underestimate the stabilization of ^3^ *A*_2*g*_ via *b*_2*g*_ occupation, and hence the state has disappeared from the possibility of the most stable state.

As a further possible explanation for the stabilization of *A*_2*g*_ as well as that of *E*_*g*_(a), we could take the energy loss due to the double occupancy on the spatially localized orbitals. Counting the double occupancies of *a*_*g*_/*e*_*g*_/*b*_2*g*_ orbitals in each state, it is 1/0/1 [*A*_2*g*_], 0/2/0 [*B*_2*g*_], 0/1/1 [*E*_*g*_(a)], and 1/1/0 [*E*_*g*_(b)], corresponding to a problem how to assign two double occupancies to each orbital. When the *U* is introduced, the system wants to avoid the double occupancy in a localized orbital, and then prefers to put it on *b*_2*g*_ with more spreading *d*_*xy*_. The states having a *d*_*xy*_ double occupancy then stabilized relatively to get ‘N-shaped’ dependence in Fig. 1. The further stabilization of *A*_2*g*_ over *E*_*g*_(a) would be explained when we look at a triplet spin pair assigned in which orbital. In *A*_2*g*_, the pair is between *d*_*zx*_ and *d*_*yz*_ which are degenerated, while in *E*_*g*_(a) they are between ${d}_{{z}^{2}}$ and one of the *d*_*zx*,*yz*_. The exchange energy gain is expected to be larger when the pair is within the degenerated orbitals, making a possible explanation for the stabilization.

## Conclusion

To conclude, we have applied DMC with the SA-CASSCF and DFT (M06, M06L and M06-2X) nodes to evaluate relative stability of the possible electronic configurations of an isolated FePc under *D*_4*h*_ symmetry. All the DMC simulations predict the ground state to be *A*_2*g*_, supporting several preceding DFT results^6, 14, 15^, though they have been regarded not well convincing because of the ambiguity about XC, as some DFT results predict different configuration^13, 15, 26^. By making comparisons between DMC and several XC, we clarified the importance of the short-range exchange effect to reproduce proper relative stability among the states. We found M06 gives the variationally best fixed node for DMC. Interestingly, within the DFT framework, the B3LYP-DFT prediction was closest to the M06-DMC one. Getting confidence about the prediction, we examined why the predicted *A*_2*g*_ is excluded from possible ground states in the recent ligand field based model^17^. The assumptions to simplify the model are identified to give unreasonably less energy gain for *A*_2*g*_ when compared with the reality. The state is found to have possible reasons for the stabilization, reducing the occupations from an unstable anti-bonding orbital, preventing double occupancies in a spatially localized orbital, and gaining exchange energy by putting a triplet spin pair into degenerated orbitals.

FePc is a typical molecule of MN_4_ macrocycles (M = transition metal), which show a promising electrochemical catalytic activity for the reduction of molecular oxygen. The binding of molecular oxygen to the MN_4_ catalyst involves binding to the *d*-orbitals of the central metal in the macrocyclic structure and will be influenced by the electronic density located on those orbitals^33^. To the best of our knowledge, this is the first work which takes into account the many-body wavefunctions to determine unambiguously the ground state of 3*d*-orbitals in FePc molecule. We believe that our results would provide useful hints about understanding the interaction of O_2_ molecules with active sites in FePc-based catalysts. As mentioned above, the short-range exchange interaction is very important to describe the relative stability of different states of FePc molecule. This provides an important insight into the choice of XC in *ab initio* molecular dynamics studies^34^ on oxygen reduction mechanism in FeN_4_ macrocycles.

## Electronic supplementary material

Supplementary Information
