# Supplementary material for: New Insight into the Ground State of FePc: A Diffusion Monte Carlo Study
Source: Sci Rep. 2017 May 17;7:2011. doi: 10.1038/s41598-017-01668-6 (PMC5435675; doi:10.1038/s41598-017-01668-6)
Supplement: Supplementary file 1 — Supplementary Information [file 41598_2017_1668_MOESM1_ESM.pdf]

Supplementary Information

New Insight into the Ground State of FePc: A Diffusion Monte Carlo Study

Tom Ichibha, Zhufeng Hou, Kenta Hongo, and Ryo Maezono

.

SA-CASSCF calculation

We performed CASSCF calculations using GAMESS (ver. 5DEC2014R1)[1,2] to generate the multi-determinant wave functions. There have been several DMC studies applied to the systems including transition metal elements with the fixed nodes generated by HF or post HF methods.[3,4] Evaluations of energy differences by DMC include the study by ROHF-DMC[5], estimating the difference between the first and second excited states of free base porphyrin, getting deviations within 0.1 eV from experimental values. Dubecky *et al.*[6] evaluated the first excitation energies of cis- (trans-) azobenzene using CASSCF-DMC, getting the deviation within 0.3 (0.01) eV. Zimmerman *et al.*[7] applied it to methylene to get deviations within 0.3 eV for differences between electronic configurations regardless of the active space sizes.

There are two options of CASSCF, state-specific CASSCF (SS-CASSCF) and state-averaged CASSCF (SA-CASSCF). In SA-CASSCF, common orbitals are applied to all the states to be evaluated while SS-CASSCF uses different ones optimized for each state individually. For the purpose to evaluate energy differences used as CASSCF-DMC, SA-CASSCF is known to be appropriate choice:[8,9] For excitation energies of an acrolein molecule, SS-CASSCF and SA-CASSCF trial nodes are compared in DMC to get the conclusion that only SA-CASSCF gives reasonable estimations, [8,9] probably due to the better error cancellations. Based on that, we generated trial nodes by SA-CASSCF.

All the elements are described as 'all-electron', getting the total number of electrons being 290. The molecular orbitals are expanded by 6-31G** Gaussian basis sets. As a nature of SA-CASSCF, we had to use the same geometries commonly for all the states. The justifying discussions are given later. The size of active space is taken as CAS(6,5) because it is almost the tractable limit within the available computational resources, especially for SA-CASSCF. We also performed SA-CASPT2 with the same conditions.

DFT calculations

We performed DFT calculations using Gaussian09[10]. We run all-electron calculations with def2QZVP basis set to compare DFT results. We used Burkatzki pseudo potentials with triple-ζ valence basis set to generate the trial nodes for DMC.[11]

To get a symmetry-adapted state for each electronic configuration in a DFT simulation, we used "guess=alter" and "scf=symm" implemented in Gaussian09: We first give an initial guess appropriate for the target state by "guess=alter", and then fix the symmetries of the all occupied orbitals during its SCF procedure by "scf=symm".

DFT+U calculations

We have performed the DFT+U calculations for FePc molecule by using a simplified version of Cococcioni and de Gironcoli[12], as implemented in QUANTUM ESPRESSO package[13]. Several different values (0, 2 and 4 eV) have been considered for Hubbard U parameter for Fe 3d orbitals. We have employed ultrasoft pseudopotentials generated with the Rappe-Rabe-Kaxiras-Joannopoulos recipe[14] to represent electron-ion interaction. The electronic exchange-correlation potential was calculated within the generalized gradient approximation (GGA) using the scheme of Perdew-Burke-Ernzerhof (PBE)[15] and the spin-polarization was taken into account. The electronic wave functions were expanded in plane waves with an energy cutoff of 35 Ry while for the charge density the energy cutoff was taken to 350 Ry. The isolated FePc molecule was simulated in a simple tetragonal cell of $27\times27\times12$ Å^3. Brillouin-zone integrations were approximated using a Γ point. The atomic positions of FePc molecule were optimized till the residual forces were less than 0.01 eV/Å.

Jastrow factor

We adopted a Jastrow factor[16] multiplied by determinant(s) to form a guiding function for DMC, imposing Kato's cusp conditions.[17] We used a function form implemented in CASINO[18] including electron-electron (u), electron-nuclei (χ), and electron-electron-nuclei (f) terms. Considering spin polarizations, u and χ (f) terms are expanded upto 8th (2nd) order of the power of inter-particle distances, getting total 144 variational parameters. Cutoff lengths for these terms are fixed as the recommended values by the implementation, and all the linear variational parameters are optimized by 'varmin-linjas' scheme.[19] For all-electron DMC, we also used the cusp-correction scheme[20] for possible electron-nuclei coalescence. All the present QMC simulations were done using CASINO (ver. 2.13)[18].

Extrapolation of time step error

For time-step error corrections in DMC,[21] we used an extrapolation scheme[22] using two time steps, $\tau_1 = \tau_{max}$ and $\tau_2 = \tau_{max}/4$. τ_{max} is taken so that it is the largest possible below which the error is proportional to τ. From the results, $E_j \pm \sigma_j$ by τ_j

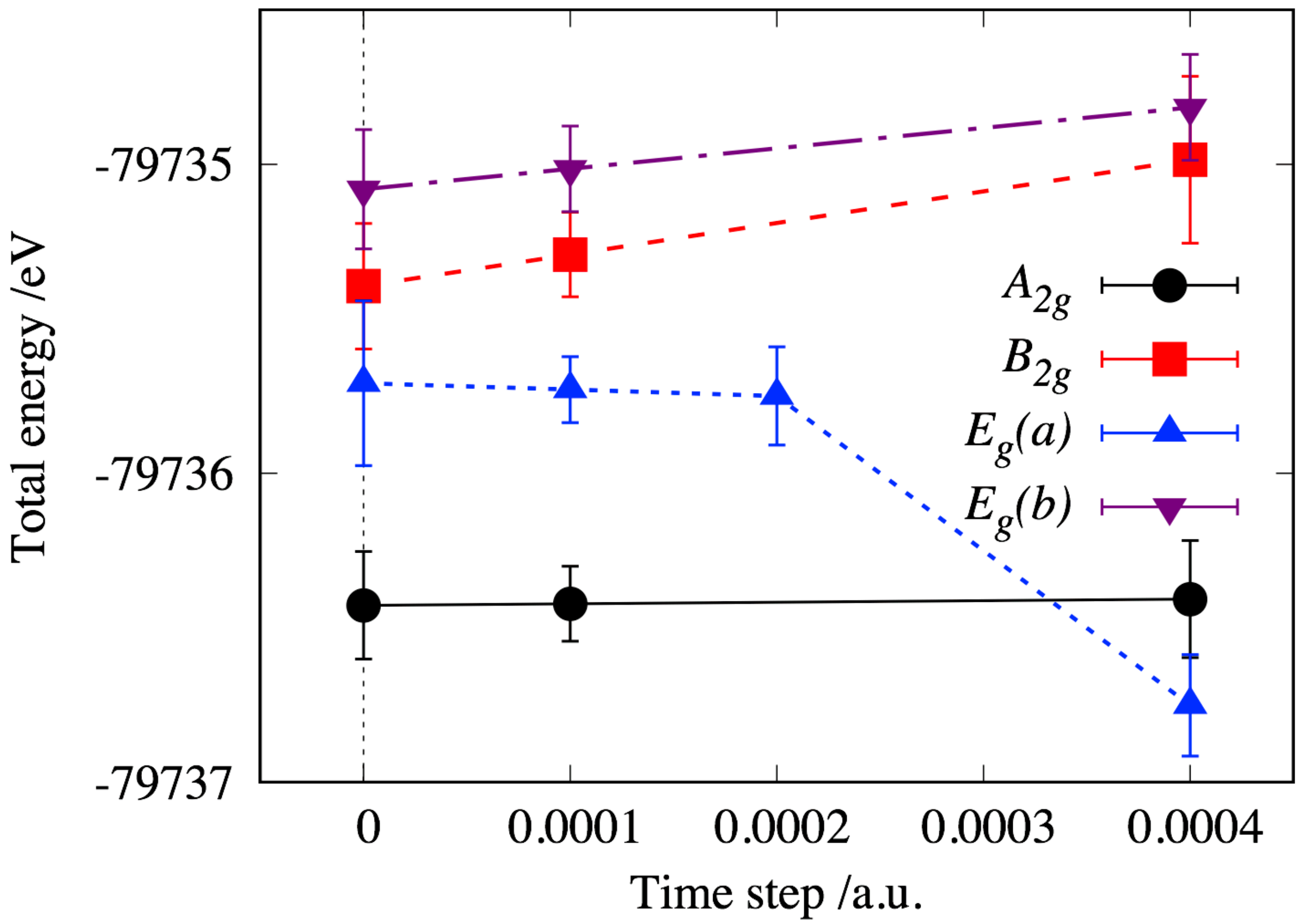

Figure S1. The extrapolation of DMC results. We can identify the ground state as A_{2g} with 1σ statistical confidence, while we cannot for other excited states. Except $E_g(a)$, linear extrapolations below $\tau_{\max} = 0.0004$ works well. For $E_g(a)$, we extrapolate using $\tau_{\max} = 0.0002$, instead.

$(j = 1,2)$, the extrapolation is evaluated as, $E(\tau \to 0) = (\tau_2 E_1 - \tau_1 E_2)\,/\,(\tau_2 - \tau_1)$ and $E(\tau \to 0) = (\tau_2 E_1 - \tau_1 E_2)\,/\,(\tau_2 - \tau_1)$ and $\sigma\tau \to 0) = \left(\sigma_1^2\tau_2^2 + \sigma_2^2\tau_1^2\right)/(\tau_2 - \tau_1)^2$.

For all electron calculations, It is proposed[22] to take $\tau_{max} < 1/(3Z^2)$ where Z is the maximum atomic number within the system. Using $Z = 26$ for the present case, we chose $\tau_{\max} = 4.0 \times 10^{-4} a.u.$ for SA-CASSCF-DMC. The choice actually proved to work except $E_g(a)$ as discussed later. The best practice is known[22] to accumulate eight times larger steps for τ_2 than that for τ_1 to minimize computational costs.

For pseudo potential calculations, $\tau_1 = 4.0 \times 10^{-3}$ and $\tau_2 = 1.0 \times 10^{-3}$ were chosen for DFT-DMC. These values are larger than those of SA-CASSCF-DMC. A necessary resolution in time step is larger in the pseudo potential case than in the all-electron because random walkers diffuse on shallower potentials.

Effects of geometry differences

As mentioned above, SA-CASSCF-DMC restricts us to use the same geometry[23] to all the states of electronic configurations. When the optimized geometry for each state largely differs from each other, this restriction could make the estimation poor. SA-CASSCF-DMC for an acrolein molecule[8] seems to be the case that it gave the overestimation of the excitation energy by ~150 meV: Though it is not explicitly stated in their paper,[8] the bond length between carbon and oxygen gets elongated by 8 %[24,25] when the system is excited. Because of the restriction, however, SA-CASSCF-DMC cannot take into account the relaxation energy gain by the elongation, and this is quite likely to be an origin of the overestimation. To examine if this matters in the present case, we evaluated the energy gains by the relaxation from the geometry used in SA-CASSCF, as tabulated in Tab. S1. It is confirmed that the gains remain within 1.3 %, corresponding to 7.7 meV which is negligibly small

Table S1. The energy gains and the bond length owing to geometry optimization. B3LYP-DFT estimations of geometry relaxation gains, ΔE, and changes in the Fe-N bond lengths ($R_{\text{Fe-N}}$). ΔE is defined as the gain when the geometries are optimized from the common structure [23] used in the present SA-CASSCF. For each E_g state, two bond lengths are given because it falls into D_{2h} from D_{4h} by the relaxation.

state	ΔE_g (meV)	$R_{\text{Fe-N}}$ (Å)
A_{2g}	−0.0	1.949
B_{2g}	−4.7 (−1.3 %)	1.946
$E_g(a)$	−1.1 (−1.2 %)	1.946/1.950
$E_g(b)$	−7.7 (−1.0 %)	1.941/1.951

compared to the statistical errors and to the energy scale in Fig. 1 in the paper. The largest relaxation is found to occur on the bond between iron and neighboring nitrogen, which is confirmed to be within 0.26 % at most. The geometry insensitivity to the occupations to be considered is, incidentally, in accordance with a report[26] that the Fe-N bonding length is mainly dominated by the occupation number of $d_{x^2-y^2}$, which is not considered here, though the conclusion is drawn from Fe porphyrin case. The insensitivity could justify the use of the same geometry to evaluate the relative stabilities among the states.

References

1. Schmidt, M. W. *et al.* General atomic and molecular electronic structure system. *J. Comput. Chem.* **14**, 1347–1363; 10.1002/jcc.540141112 (1993).
2. Gordon, M.S. & Schmidt, M.W. Theory and Applications of Computational Chemistry (ed. Dykstra C.E. *et al.*) 1167-1189 (Elsevier, 2005).
3. Hongo, K. & Maezono, R. A benchmark quantum Monte Carlo study of the ground state chromium dimer. *Int. J. Quant. Chem.* **112**, 1243–1255; 10.1002/qua.23113 (2012).
4. Koseki, J., Maezono, R., Tachikawa, M., Towler, M. D. & Needs, R. J. Quantum Monte Carlo study of porphyrin transition metal complexes. *J. Chem. Phys.* **129**, 085103; 10.1063/1.2966003 (2008).
5. Aspulu-Guzik, A., Akramine, O. E., Grossman, J. C. & Lester, W. A. Quantum Monte Carlo for electronic excitations of free-base porphyrin. *J. Chem. Phys.* **120**, 3049 3050; 10.1063/1.1646356 (2004).
6. Dubecký, M., Derian, R., Mitas, L. & Štich, I. Ground and excited electronic states of azobenzene: A quantum monte carlo study. *J. Chem. Phys* **133**, 244301; 10.1063/1.3506028 (2010).
7. Zimmerman, P. M., Toulouse, J., Zhang, Z., Musgrave, C. B. & Umrigar, C. J. Excited states of methylene from quantum monte carlo. *J. Chem. Phys* **131**, 124103; 10.1063/1.3220671 (2009).
8. Bouabça, T., Ben Amor, N., Maynau, D. & Caffarel, M. A study of the fixed-node error in quantum Monte Carlo calculations of electronic trans itions: The case of the singlet $n \to \pi^*$ (CO) transition of the acrolein. *J. Chem. Phys.* **130**, 114107; 10.1063/1.3086023 (2009).
9. Toulouse, J., Caffarel, M., Reinhardt, P., Hoggan, P. E. & Umrigar, C. J. Advances in the Theory of Quantum Systems in Chemistry and Physics (ed. Hoggan, E.P. *et al.*) 343-351 (Springer Netherlands, 2012).
10. Frisch, M.J. *et al.*, Gaussian 09 Revision D.01. Gaussian Inc. Wallingford CT 2009.
11. Burkatzki, M., Filippi, C. Dolg, M.J. Energy-consistent pseudopotentials for quantum Monte Carlo calculations. *J. Chem. Phys.* **126**, 234105; 10.1063/1.2741534 (2007).
12. Cococcioni, M. & de Gironcoli, S. Linear response approach to the calculation of the effective interaction parameters in the LDA + U method. *Phys. Rev. B* **71**, 035105; 10.1103/PhysRevB.71.035105 (2005).
13. Giannozzi, P. *et al.* QUANTUM ESPRESSO: a modular and open-source software project for quantum simulations of materials. *J. Phys.: Condens. Mat.* **21**, 395502; 10.1088/0953-8984/21/39/395502 (2009).
14. Rappe, A.M., Rabe, K.M., Kaxiras, E. & Joannopoulos, J.D. Optimized Pseudopotentials. *Phys. Rev. B* **41**, 1227; 10.1103/PhysRevB.41.1227 (1990).
15. Perdew, J.P., Burke, K. & Ernzerhof, M. Generalized Gradient Approximation Made Simple. *Phys. Rev. Lett.* **77**, 3865; 10.1103/PhysRevLett.77.3865 (1996).

16. Drummond, N. D., Towler, M. D. & Needs, R. J. Jastrow correlation factor for atoms, molecules, and solids. *Phys. Rev. B* **70**, 235119; 10.1103/PhysRevB.70.235119 (2004).

17. Kato, T. On the eigenfunctions of many-particle systems in quantum mechanics. *Communications on Pure and Applied Mathematics* **10**, 151–177; 10.1002/cpa.3160100201 (1957).

18. Needs, R., Towler, M., Drummond, N. & Ríos, P. L. Continuum variational and diffusion quantum Monte Carlo calculations. *J. Phys. Condens. Matter* **22**, 023201; 10.1088/0953-8984/22/2/023201 (2010).

19. Drummond, N. D. & Needs, R. J. Variance-minimization scheme for optimizing Jastrow factors. *Phys. Rev. B* **72**, 085124; 10.1103/PhysRevB.72.085124 (2005).

20. Ma, A., Towler, M. D., Drummond, N. D. & Needs, R. J. Scheme for adding electron-nucleus cusps to Gaussian orbitals. *J. Chem. Phys.* **122**, 224322; 10.1063/1.1940588 (2005).

21. Foulkes, W. M. C., Mitas, L., Needs, R. J. & Rajagopal, G. Quantum Monte Carlo simulations of solids. *Rev. Mod. Phys.* **73**, 33; 10.1103/RevModPhys.73.33 (2001).

22. Lee, R. M., Conduit, G. J., Nemec, N., Ríos, P.L. & Drummond, N. D. Strategies for improving the efficiency of quantum Monte Carlo calculations. *Phys. Rev. E* **83**, 066706; 10.1103/PhysRevE.83.066706 (2011).

23. Sumimoto, M., Kawashima, Y. Hori, K. & Fujimoto, H. Theoretical investigation of the molecular and electronic structures and excitation spe ctra of iron phthalocyanine and its derivatives, FePc and $FePcL_n$ (L=Py, CN^- ; n=1,2). *Dalton Trans.* 5737–5746; 10.1039/B823309H (2009).

24. Inuzuka, K. Near Ultraviolet Absorption Spectra of Acrolein and Crotonaldehyde. *Bull. Chem. Soc. Jap.* **33**, 678–680; 10.1246/bcsj.33.678 (1960).

25. Blom, C. E., Grassi, G. & Bauder, A. Molecular Structure of s-cis- and s-trans-Acrolein Determined by Microwave Spectrosco py. *J. Am. Chem. Soc.* **106**, 7427–7431; 10.1021/ja00336a022 (1984).

26. Choe, Y.-K., Hashimoto, T., Nakano, H. & Hirao, K. Theoretical study of the electronic ground state of iron(II) porphine. *Chem. phys. Lett.* **295**, 380–388; 10.1016/S0009-2614(98)00986-5 (1998).
